# Outcomes of High-Flow Nasal Cannula Vs. Nasal Continuous Positive Airway Pressure in Young Children With Respiratory Distress: A Systematic Review and Meta-Analysis

**DOI:** 10.3389/fped.2021.759297

**Published:** 2021-11-05

**Authors:** Xueqin Zhao, Qiaozhi Qin, Xian Zhang

**Affiliations:** Department of Pediatric, Northern Jiangsu People's Hospital, Yangzhou, China

**Keywords:** continuous positive airway pressure (CPAP), high-flow nasal cannula (HFNC), acute respiratory distress, young children, bronchiolitis, pneumonia, meta-analysis

## Abstract

**Background:** Continuous positive airway pressure (CPAP) has been associated with a lower risk of treatment failure than high-flow nasal cannula (HFNC) in pediatric patients with respiratory distress and severe hypoxemia. However, the publication of new trials on children younger than 2 years warrants a review and updated meta-analysis of the evidence.

**Methods:** We conducted a systematic search in the PubMed, Scopus, and Google scholar databases for randomized controlled trials (RCTs) in pediatric patients with acute respiratory distress that examined outcomes of interest by the two usual management modalities (CPAP and HFNC). We used pooled adjusted relative risks (RRs) to present the strength of association for categorical outcomes and weighted mean differences (WMDs) for continuous outcomes.

**Results:** We included data from six articles in the meta-analysis. The quality of the studies was deemed good. Included studies had infants with either acute viral bronchiolitis or pneumonia. Compared to CPAP, HFNC treatment carried a significantly higher risk of treatment failure [RR, 1.45; 95% CI, 1.06 to 1.99; *I*^2^ = 0.0%, *n* = 6]. Patients receiving HFNC had a lower risk of adverse events, mainly nasal trauma [RR, 0.30; 95% CI, 0.14 to 0.62; *I*^2^ = 0.0%, *n* = 2] than the others. The risk of mortality [RR, 3.33; 95% CI, 0.95, 11.67; *n* = 1] and need for intubation [RR, 1.69; 95% CI, 0.97, 2.94; *I*^2^ = 0.0%, *n* = 5] were statistically similar between the two management strategies; however, the direction of the pooled effect sizes is indicative of a nearly three times higher mortality and two times higher risk of intubation in those receiving HFNC. We found no statistically significant differences between the two management modalities in terms of modified woods clinical asthma score (M-WCAS; denoting severity of respiratory distress) and hospitalization length (days). Patients receiving HFNC had the time to treatment failure reduced by approximately 3 h [WMD, −3.35; 95% CI, −4.93 to −1.76; *I*^2^ = 0.0%, *n* = 2] compared to those on CPAP.

**Conclusions:** Among children with respiratory distress younger than 2 years, HFNC appears to be associated with higher risk of treatment failure and possibly, an increased risk of need for intubation and mortality. Adequately powered trials are needed to confirm which management strategy is better.

## Introduction

Acute bronchiolitis and pneumonia are major respiratory failure causes in children and infants (1, 2). Approximately 15–20% of affected children require respiratory support and intensive care due to a rapid emergence of respiratory distress (3, 4). Pneumonia and bronchiolitis are major causes of death in children under 5 years; and, approximately 1–1.5 million children die annually of pneumonia worldwide (this number includes ~200,000 cases with bronchiolitis) (3, 5). The World Health Organization and the American Academy of Pediatrics (AAP) both recommend oxygen supplementation at an arterial pulse oximetry (SpO_2_) lower than 90% because oxygen supply in children with acute lower respiratory tract infection is associated with reduced mortality (6–8).

Different modalities for oxygen supplementation in children exist. The standard flow oxygen therapy (through a standard nasal cannula) provides oxygen without the need for humidification when the oxygen flow is either low (i.e., 1–2 L/min) or the room air has high humidity (6, 9). On the other hand, high flow rates usually require humidification due to the drying effect of non-humidified cold oxygen on nasal secretions and the respiratory mucosa (10). High-flow nasal cannula (HFNC) oxygen therapy delivers warm and humidified oxygen at a higher flow than the normal inspiratory flow (11). Studies have suggested its usefulness for improving oxygenation and alleviating the requirement for mechanical ventilation in children with respiratory distress (12–14). The nasal continuous positive airway pressure (CPAP) is another management modality limited in resource-constrained settings and usually requiring technical skills along with adequate maintenance. Continuous positive airway pressure combines supplemental oxygen with a positive end-expiratory pressure (PEEP); it has been shown to reduce the ventilation need duration and the overall hospitalization length in children with severe bronchiolitis (15, 16).

Both CPAP and HFNC are high flow systems and are capable of generating PEEP (17). High-flow nasal cannula is considered to be a less invasive procedure than CPAP, better tolerated and comparatively easy to perform (18). This probably makes HFNC a preferred procedure of choice in young children. One of the important differences between these two procedures is that CPAP employs an integrated pressure release valvular system, whereas in HFNC, the release of pressure is via the leak at the nares-prong interface and through the mouth (17). The lack of the ability to regulate the pressure delivered to the airways in HFNC may run the risk of delivering high pressures at high flow rates if the leak is compromised (19, 20). High-flow nasal cannula is thought to work through increasing the oxygen fraction in the alveoli by washout of the nasopharyngeal dead space, reducing the inspiratory resistance, improvement of airway conductance and by providing an end-distending pressure to the lungs (21–23). On similar lines, CPAP decreases the inspiratory resistance, reduces atelectasis, reduces alveolar resistance, increases surface area of alveoli, and enhances ventilation and perfusion (V/Q) matching through PEEP (24, 25).

Studies, both observational and randomized controlled trials (RCTs), have been published previously that compared outcomes among children receiving HFNC and CPAP. A retrospective record-based study to compare CPAP with HFNC among infants with acute bronchiolitis found no significant difference in length of hospital stay, respiratory rate, PaCO_2_, FiO_2_, or duration of oxygen supply among the two groups (26). The authors concluded no difference between the two modalities in the management of children with severe bronchiolitis. Another observational study compared the two modalities among children with moderate to severe respiratory distress (27). The study found no significant difference with respect to respiratory rate and arterial oxygen saturation between the two groups. Around one-quarter (26%) of children in HFNC groups required escalation of respiratory support as against around one-fifth (18%) in CPAP group (P of 0.27). A retrospective study by Pederson et al. in a sample of 49 children with median age of 1.9 months found no difference in length of treatment, hospital stay, complication rate and transmission to intensive care unit between the CPAP and HFNC groups (17). However, CPAP was more effective than HFNC in decreasing respiratory rate and FiO_2_. RCTs among preterm neonates have indicated that HFNC had effects similar or inferior to CPAP (13, 28, 29).

A meta-analysis by Luo et al. compared the outcomes in pediatric patients with respiratory distress after HFNC and CPAP (30), but included data from four studies only. The findings indicate that HFNC had an increased risk of treatment failure and a lower risk of nasal trauma compared with CPAP. No significant differences were found in intubation rates and mortality between the HFNC and CPAP groups. Another recent review by Lin et al., involving 2,121 children from nine randomized trials found a significant increase of the incidence of treatment failure [Relative risk (RR) of 1.61] in children receiving HFNC compared with those receiving CPAP (31). However, the review noted no other significant difference in other outcomes such as length of hospital stay, incidence of need for intubation, respiratory rate, SpO_2_ and adverse events between the two groups. The publication of new RCTs warrants an update of the previous evidence on this issue. Therefore, we compared HFNC and CPAP outcomes in pediatric patients with respiratory distress by pooling data from RCTs and performing a meta-analysis.

## Materials and Methods

### Search Strategy

Our study processes complied with the PRISMA (Preferred Reporting Items for Systematic Reviews and Meta-analyses) guidelines (32). We carried out a thorough systematic search of English language papers published until 15^th^ April 2021 on electronic search engines (PubMed, Scopus, and Google academic databases). We used medical subject headings (MeSH) terminology as well as free text words. Supplementary Table 1 presents the search strategy details. The literature search aimed at identifying studies on pediatric populations with acute respiratory distress that compared outcomes of interest after treatments with either CPAP or HFNC. The primary outcomes of interest were treatment failure, intubation need, mortality, and any adverse events. Secondary outcomes were treatment duration, respiratory distress severity, hospitalization length, time to treatment failure, respiratory rate, and blood gas parameters (SpO_2_, PaCO_2_, PaO_2_, and FiO_2_).

### Selection Criteria and Methods

Two subject experts from the team reviewed the studies in the initial search results after removing duplicates; they initially screened the titles and abstracts and then reviewed the full text of candidate studies. Disagreements in the inclusion of studies were resolved through discussions between the authors. We only included those studies that fulfilled the inclusion criteria in the meta-analysis. We also checked the reference list of the studies included to identify additional literature.

#### Inclusion Criteria

We considered only RCTs for inclusion. The trials had to include pediatric patients with acute respiratory distress and they had to have examined the outcomes of interest after use of CPAP or HFNC.

#### Exclusion Criteria

We excluded studies with other designs (cohorts, cross-sectional, or case-report studies, or reviews). In addition, we excluded studies that failed to provide data on the outcomes of interest or that did not include comparative findings between CPAP and HFNC.

### Data Extraction and Quality Assessment

Two study authors separately extracted the relevant data from the included studies onto a pretested data extraction sheet. The extracted data included a study identifier (the name of the first author along with the publication year), the study setting (the country where the study was carried out), and the study design, subject characteristics, overall sample size, and main findings. The methodological assessment was done independently by two authors using the Cochrane assessment tool (33).

### Statistical Analysis

We used STATA version 16.0 to perform the meta-analysis. We expressed effect sizes as pooled RRs with 95% confidence intervals (CIs) for categorical outcomes and weighted mean differences (WMDs) for continuous outcomes. We calculated *I*^2^ as a measure of heterogeneity; moreover, we applied a random effects model in instances where the value of *I*^2^ exceeded 40% (34). We set the significance value at a *p*-value lower than 0.05. Egger's test was used to assess publication bias (19). For the primary outcomes, we also did a subgroup analysis based on the clinical diagnosis i.e., acute viral bronchiolitis and pneumonia.

## Results

### Selection of Articles, Study Characteristics, and Quality of Included Studies

**Figure 1:**
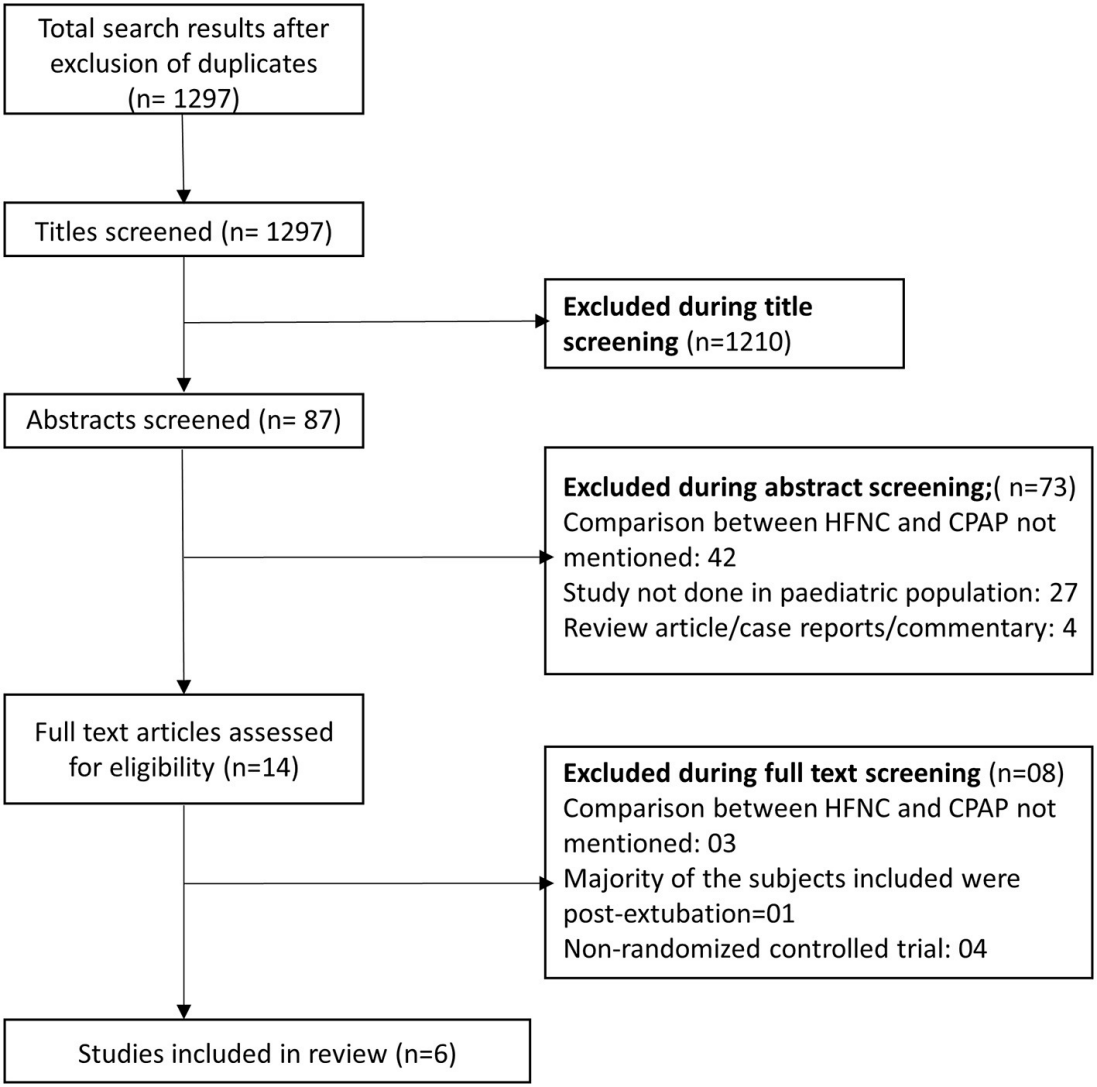
Selection process of the studies included in the review.

We obtained 1,297 citations after our database search and the removal of duplicates (Figure 1). We excluded 1,210 studies after title screening. Then, we excluded 73 studies after reading of the abstract. We reviewed the remaining 14 papers in detail and used data from the 6 articles fitting all our inclusion criteria in our meta-analysis (35–40). Table 1 presents details of the included studies. All the included studies were RCTs. Each study was conducted in a different country (Denmark, United States, China, Bangladesh, France, and India). All the six studies were done in infants. Five studies were done in infants younger than 6 months but none of them included neonates (i.e., those within 28 days of age). In four studies, the primary diagnosis in included infants was acute viral bronchiolitis (35, 36, 39, 40). In remaining two studies, the primary clinical condition was pneumonia (37, 38). Supplementary Table 2 shows the results of the quality evaluation of the included studies. We deemed the quality of the included studies as good. All the studies reported random sequence generation and allocation concealment. Blinding could not be established as all the included studies were open label trials and both management techniques (HFNC and CPAP) are widely used in the clinical practice and readily recognized by clinicians. We did not notice any major bias in any of the studies.

**Table 1:** Characteristics of the studies included in the meta-analysis.

**Reference**	**Study design**	**Country**	**Participant characteristics**	**Devices and process used**	**Sample size**	**Key outcome (HFNC vs. CPAP)**
Vahlkvist et al. (35)	Prospective open randomized trial	Denmark	Infants and young children with bronchiolitis, median age of around 2 months, mean weight of 5.2 kg, and 90% RSV positive	CPAP: binasal prong with a Benveniste valve connected to a humidifier was used. The initial flow was 12–14 L/min. HFNC: Optiflow Junior was used. Three sizes of nasal prongs were used, according to the weight of the child. The initial flow was 2 L/kg/min. In both systems, flow could be increased to a maximum of 15/L/min and oxygen supply delivered as needed to maintain a SpO_2_ above 92%.	50 (22 with high flow oxygenation therapy, HFNC and 28 with continuous positive airway pressure, CPAP)	**Mean (SD) treatment duration (in)**: 95 (27.7) vs. 70 (39) **Treatment failure:** RR 0.64 (95% CI: 0.13, 3.16) **Mean (SD) modified woods clinical asthma score (M-WCAS) at 48 h of treatment**: 2 (0.9) vs. 1.5 (1.0) **Neonatal infant pain score (mean, SD) at 48 h of treatment**: 0.4 (1.0) vs. 0.5 (1.0) **Mean (SD) respiratory rate at 48 h of treatment:** 45 (10) vs. 43 (12) **Mean (SD) PaCO**_**2**_ **(mm Hg) at 48 h of treatment:** 46.5 (1.1) vs. 47.3 (1.5) **Mean (SD) FiO**_**2**_ **(%) at 48 h of treatment:** 25 (4.6) vs. 22 (5.1)
Cesar et al. (36)	Randomized controlled trial	United States	Infants and young children with critical bronchiolitis, median age of 2.7 months, mean weight of 5.7 kg, and 89% RSV positive	CPAP group: properly sized soft anatomically curved nasal prongs. CPAP was generated through a Dräger Evita 4 ventilator outfitted with a heated humidifier. CPAP was set at 6 cm H_2_O for all patients. HFNC group: nasal cannula sized to occlude no more than 50% of the cross-sectional area of the nostrils. HFNC support provided through a dedicated hollow fiber heated humidified system with a disposable circuit. Flow was titrated up to a maximum of 1.5 L/kg/min, as needed, based on clinical assessment. Both experimental groups: fraction of inspired oxygen (FiO_2_) was adjusted to achieve a SpO_2_ >93%.	63 (35 with high flow oxygenation therapy, HFNC and 28 with continuous positive airway pressure, CPAP)	**Treatment failure:** RR 1.10 (95% CI: 0.57, 2.12) **Need for intubation**: RR 1.79 (95% CI: 0.61, 5.24) **Mean (SD) modified woods clinical asthma score (M-WCAS) at end of treatment**: 5 (0.33) vs. 4.5 (0.17) **Mean (SD) treatment duration (in h)**: 67 (11.2) vs. 56.12 (7.86) **Mean (SD) respiratory rate at end of treatment:** 48.36 (11.2) vs. 45.22 (13.9) **Mean (SD) FiO**_**2**_ **(%) at end of treatment:** 40 (2.0) vs. 40 (3.0) **Mean (SD) SPO**_**2**_ **(%) at end of treatment**: 97 (0.58) vs. 97 (0.38) **Mean (SD) time to treatment failure (in h):** 15.2 (2.1) vs. 18.8 (5.3) **Mean (SD) length of hospital stay (in days):** 9 (0.83) vs. 8 (0.67)
Liu et al. (37)	Randomized controlled trial	China	Infants and young children mild to moderate respiratory failure due to pneumonia, median age of 3 months, 44% females, mean weight of 6.0 kg, and 65% RSV positive	CPAP group: the initial parameter was set at 50–60% oxygen concentration, the pressure was set at 4–6 cm H_2_O, and the flow rate of oxygen supply was set at 5–10 L/min to maintain the transcutaneous oxygen saturation ≥92–94%. HFNC group: received Airvo2 type warm humidification high flow double chamber nasal oxygen therapy ventilator. The initial parameter was set at 50–60% oxygen concentration, and the inhaled oxygen flow was set at 2 L/kg/min to a limit of 20 L/min to maintain the transcutaneous oxygen saturation ≥92–9%.	84 (43 with high flow oxygenation therapy, HFNC and 41 with continuous positive airway pressure, CPAP)	**Treatment failure:** RR 1.43 (95% CI: 0.43, 4.70) **Need for intubation**: RR 1.41 (95% CI: 0.45, 4.20) **Mean (SD) length of hospital stay (in days):** 8 (0.33) vs. 8 (0.33) **Adverse events:** RR 0.17 (95% CI: 0.04, 0.74) **Mean (SD) respiratory rate at 48 h of treatment:** 51.5 (8.2) vs. 51.5 (5.7) **Mean (SD) SPO**_**2**_ **(%) at 48 h of treatment**: 95 (0.33) vs. 96 (0.33) **Mean (SD) PaCO**_**2**_ **(mm Hg) at 48 h of treatment:** 40.5 (1.83) vs. 41 (1.33) **Mean (SD) PaO**_**2**_ **(mm Hg) at 48 h of treatment:** 91.5 (2.13) vs. 96 (6.87) **Use of sedatives**: RR 0.48 (95% CI: 0.32, 0.71)
Chisti et al. (38)	Randomized controlled trial	Bangladesh	Infants and young children with severe pneumonia and hypoxemia, median age of 7 months, 12% with bacteraemia	Bubble CPAP system: constructed using standard nasal oxygen prongs, tubing used for administration of intravenous fluids and a water-filled, transparent shampoo bottle. Gas flow was provided by oxygen concentrators. The positive end-expiratory pressure provided by CPAP was started at 5 cm H_2_O and increased up to 10 cm H_2_O if the child was not responding. HFNC: oxygen concentrator was used to provide a mixture of air and oxygen of 2 L per kg of bodyweight per min up to a maximum of 12 L/min. The high-flow oxygen was passed through a room-temperature water humidifier and delivered via nasal oxygen prongs	158 (79 with high flow oxygenation therapy, HFNC and 79 with continuous positive airway pressure, CPAP)	**Treatment failure:** RR 2.00 (95% CI: 0.72, 5.59) **Need for intubation**: RR 2.00 (95% CI: 0.72, 5.59) **Mortality:** RR 3.33 (95% CI: 0.95, 11.66) **Mean (SD) length of hospital stay (in days):** 5 (0.67) vs. 5 (0.65)
Milesi et al. (39)	Randomized controlled trial	France	Infants and young children with severe acute viral bronchiolitis, mean age of 40 days, mean weight of 4 kg, 88% with RSV	Two different systems used to generate nCPAP: the Infant Flow Ventilator and the FlexiTrunk infant interface connected to ventilator CPAP setups. Positive continuous pressure was set at +7 cmH_2_O. HFNC: device used was the Optiflow. Flow was delivered at 2 L/kg/min, with the device equipped with a pressure release valve set at 45 cmH_2_O. In both groups, FiO_2_ was titrated in order to achieve a SpO_2_ of 94–97% and the humidifier was auto set at 37°C.	142 (71 with high flow oxygenation therapy, HFNC and 71 with continuous positive airway pressure, CPAP)	**Treatment failure:** RR 1.64 (95% CI: 1.08, 2.48) **Need for intubation**: RR 1.67 (95% CI: 0.41, 6.71) **Mean (SD) time to treatment failure (in h):** 6.7 (5.7) vs. 9.7 (8.8) **Mean (SD) length of hospital stay (in days):** 6.2 (6) vs. 7.5 (13) **Mean (SD) treatment duration (in h)**: 98.3 (100.6) vs. 72.9 (46.3) **Mean (SD) respiratory rate at end of treatment:** 58 (21) vs. 46 (11) **Mean (SD) modified woods clinical asthma score (M-WCAS) at end of treatment**: 4 (1) vs. 4 (1.0)
Sarkar et al. (40)	Randomized controlled trial	India	Infants with acute bronchiolitis, median age of 3.4 months	CPAP: CPAP started at 4 cm H_2_O and increased up to a maximum of 8 cm H_2_O. Nasal prong or nasal mask of appropriate size which was snugly fitted and produces minimum leak and maximum comfort was used as interface. HFNC: provided continuously through large bore binasal prongs, with a gas flow rate of 2 L/kg/min for the children <10 kg and for children >10 kg, 2 L/kg/min for the first 10 kg + 0.5 L/kg/min for each kg above that and FiO_2_ of 0.4 at initiation.	31 (15 with high flow oxygenation therapy, HFNC and 16 with continuous positive airway pressure, CPAP)	**Treatment failure:** RR 1.07 (95% CI: 0.07, 15.57) **Need for intubation**: RR 1.07 (95% CI: 0.07, 15.57) **Mean (SD) length of hospital stay (in days):** 5 (1.6) vs. 5 (1.79) **Mean (SD) treatment duration (in h)**: 86.4 (15.12) vs. 91.2 (19.2) **Adverse events (nasal injury):** RR 0.36 (95% CI: 0.15, 0.86) **Mean (SD) SPO**_**2**_ **(%) at 48 h of treatment**: 97.4 (1.22) vs. 97 (1.36) **Mean (SD) PaO**_**2**_ **(mm Hg) at 48 h of treatment:** 94.8 (5.14) vs. 98.4 (6.74) **Mean (SD) PaCO**_**2**_ **(mm Hg) at 48 h of treatment:** 40 (4.4) vs. 37.2 (4.09)
				The fraction of oxygen in the gas flowing in the system was adjusted to maintain a SpO_2_ of 94% or more.		**Mean (SD) respiratory rate at 48 h of treatment:** 36.5 (5.17) vs. 36.2 (2.40) **Mean (SD) modified woods clinical asthma score (M-WCAS) at 48 h of treatment:** 2.1 (0.86) vs. 2.2 (0.68)

### Primary Outcome Findings

**Figure 2:**
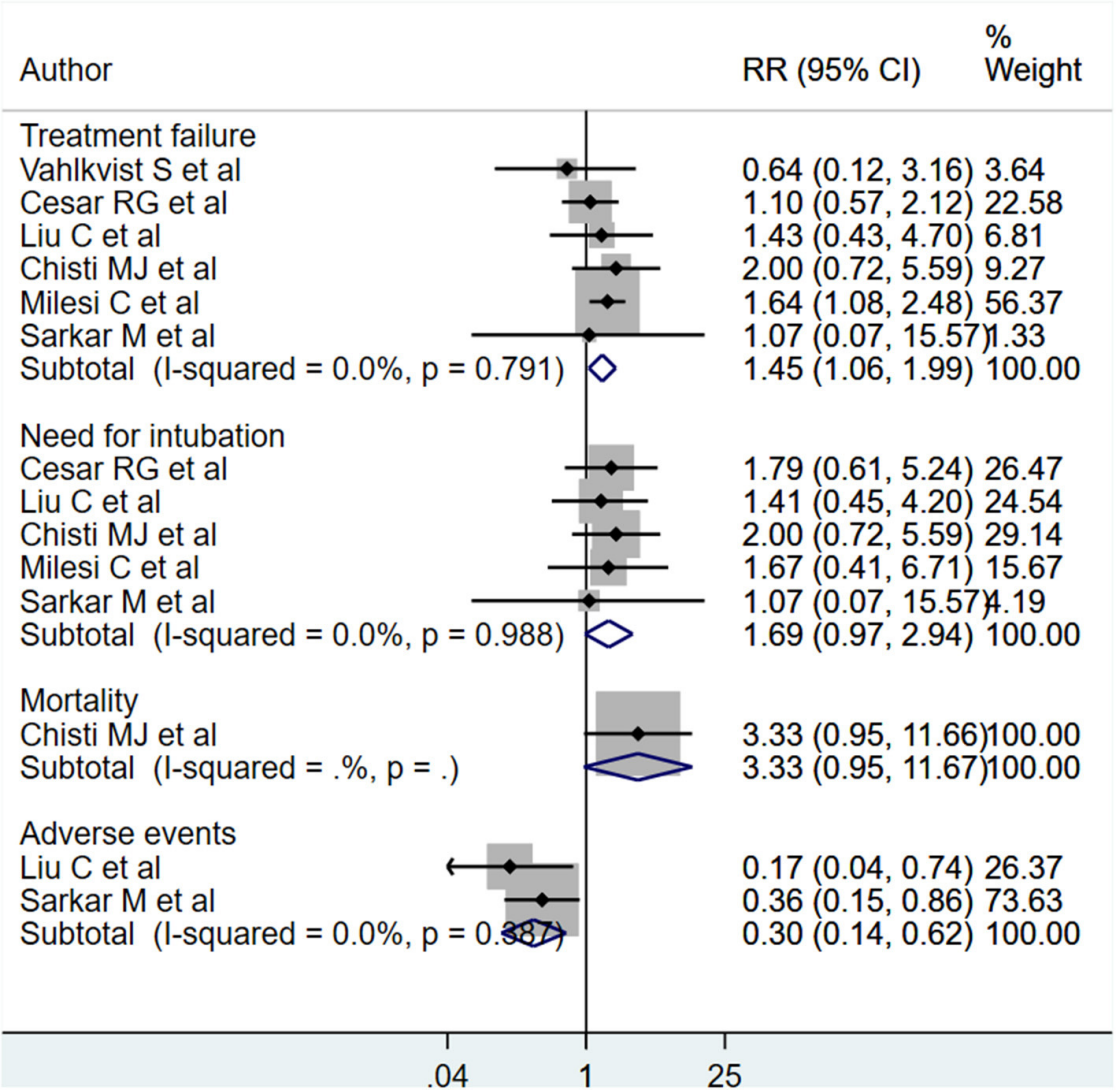
Effects of HFNC vs. CPAP on treatment failure, need for intubation, mortality and adverse events.

The HFNC treatment carried significantly higher risks of treatment failure [RR, 1.45; 95% CI, 1.06 to 1.99; *I*^2^ = 0.0%, *n* = 6]. Although the pooled effect sizes for risk of need for intubation [RR, 1.69; 95% CI, 0.97 to 2.94; *I*^2^ = 0.0%, *n* = 5] and mortality [RR, 3.33; 95% CI, 0.95 to 11.67; *n* = 1] did not achieve statistical significance, the direction of the effect sizes tended toward significance and indicated a nearly three times higher risk for mortality and two times higher risk of need for intubation in those receiving HFNC, compared to CPAP treatment (Figure 2). It should however be noted that there was only one study reporting on mortality. Patients receiving HFNC had lower risks of adverse events [RR, 0.30; 95% CI, 0.14, 0.62; *I*^2^ = 0.0%, *n* = 2] (the most common adverse event was nasal trauma). Egger's test did not indicate the presence of publication bias (*P* = 0.31 for treatment failure, *P* = 0.47 for need for intubation, and *P* = 0.29 for adverse events).

We did a subgroup analysis based on the primary diagnosis i.e., acute viral bronchiolitis or pneumonia. The findings are presented in Table 2. The increased risk of treatment failure in children receiving HFNC, compared to those receiving CPAP, was noted only in infants with acute viral bronchiolitis [RR, 1.40; 95% CI, 1.02 to 1.97; *I*^2^ = 0.0%, *n* = 4]. The reduced risk of adverse events (i.e., nasal trauma) was noted in infants with acute viral bronchiolitis [RR, 0.36; 95% CI, 0.15 to 0.86; *n* = 1] as well as pneumonia [RR, 0.17; 95% CI, 0.04 to 0.73; *n* = 1].

**Table 2:** Findings of subgroup analysis for the primary outcomes based on clinical diagnosis.

	**Pooled effect size (relative risk; RR)**	
	**(95% confidence interval)**	
	**Acute viral bronchiolitis**	**Pneumonia**
Treatment failure	1.40 (1.02, 1.97; *N* = 4)^*^	1.74 (0.80, 3.78; *N* = 2)
Need for intubation	1.67 (0.74, 3.76; *N* = 3)	1.70 (0.80, 3.63; *N* = 2)
Adverse events	0.36 (0.15, 0.86; *N* = 1)^*^	0.17 (0.04, 0.73; *N* = 1)^*^

* *Denotes statistical significance at P < 0.05; subgroup analysis for mortality not done as only one study reported this outcome*.

### Secondary Outcome Findings

**Figure 3:**
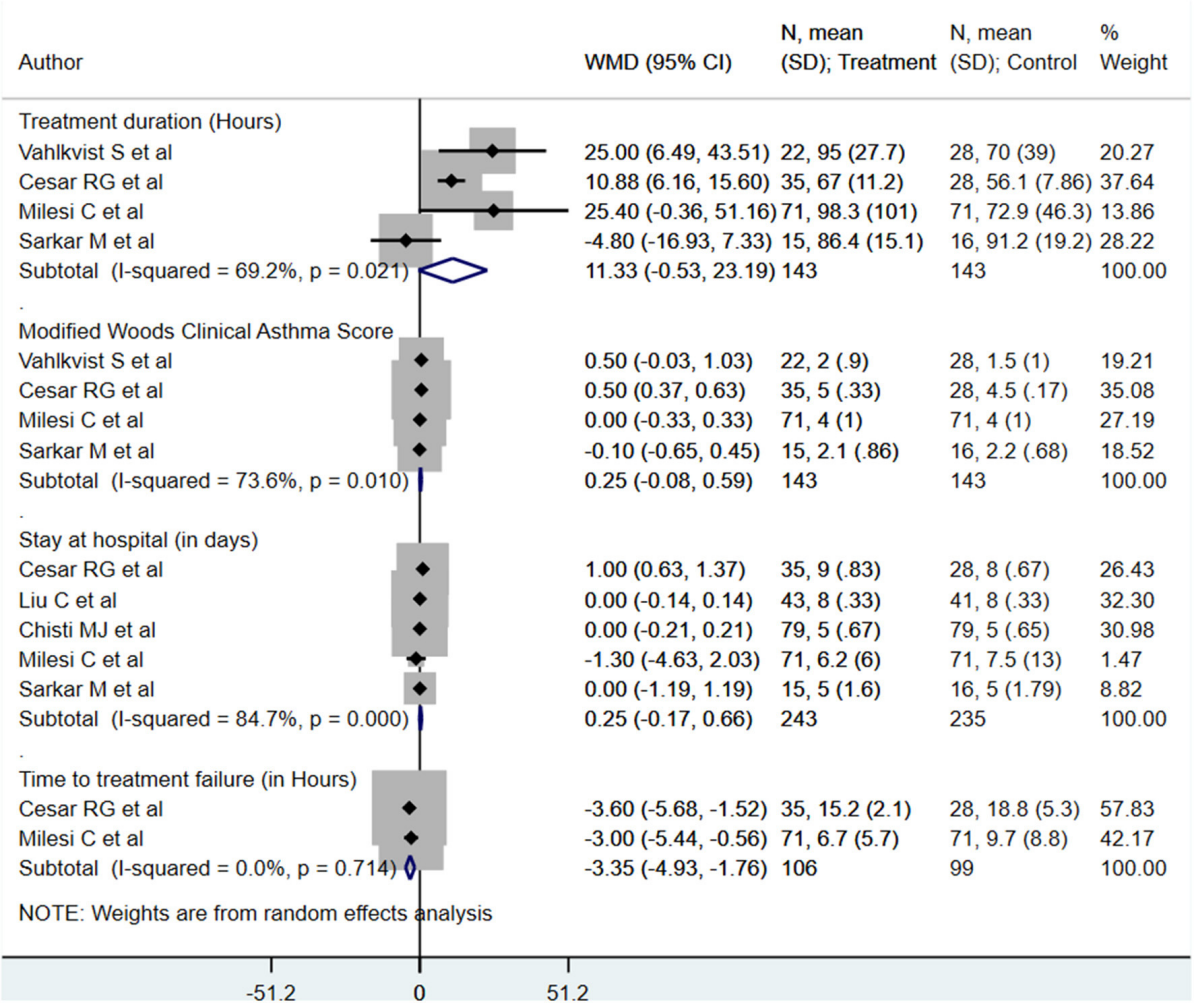
Effects of HFNC vs. CPAP on primary treatment duration, modified Woods clinical asthma score, hospitalization length, and time to treatment failure.

We found no statistically significant differences in modified Woods clinical asthma score (M-WCAS; denoting severity of respiratory distress) [WMD, 0.25; 95% CI, −0.08 to 0.59; *I*^2^ = 73.6%, *n* = 4], or hospitalization length (days) [WMD, 0.25; 95% CI, −0.17 to 0.66; *I*^2^ = 84.7%, *n* = 5] between the two management modalities (Figure 3). Patients receiving HFNC had the time to treatment failure reduced by approximately 3 h [WMD, −3.35; 95% CI, −4.93, −1.76; *I*^2^ = 0.0%, *n* = 2] as compared to those on CPAP (Figure 3). For the primary treatment duration (in h), although the pooled effect size was not statistically significant, the direction of the finding was suggestive of an increased treatment duration in those that received HFNC [WMD, 11.33; 95% CI, −0.53 to 23.19; *I*^2^ = 69.2%, *n* = 4]. Egger's test results did not indicate the presence of publication bias (*P* = 0.38 for primary treatment duration; *P* = 0.24 for M-WCAS, *P* = 0.77 for hospitalization length, *P* = 0.35 for time to treatment failure).

**Figure 4:**
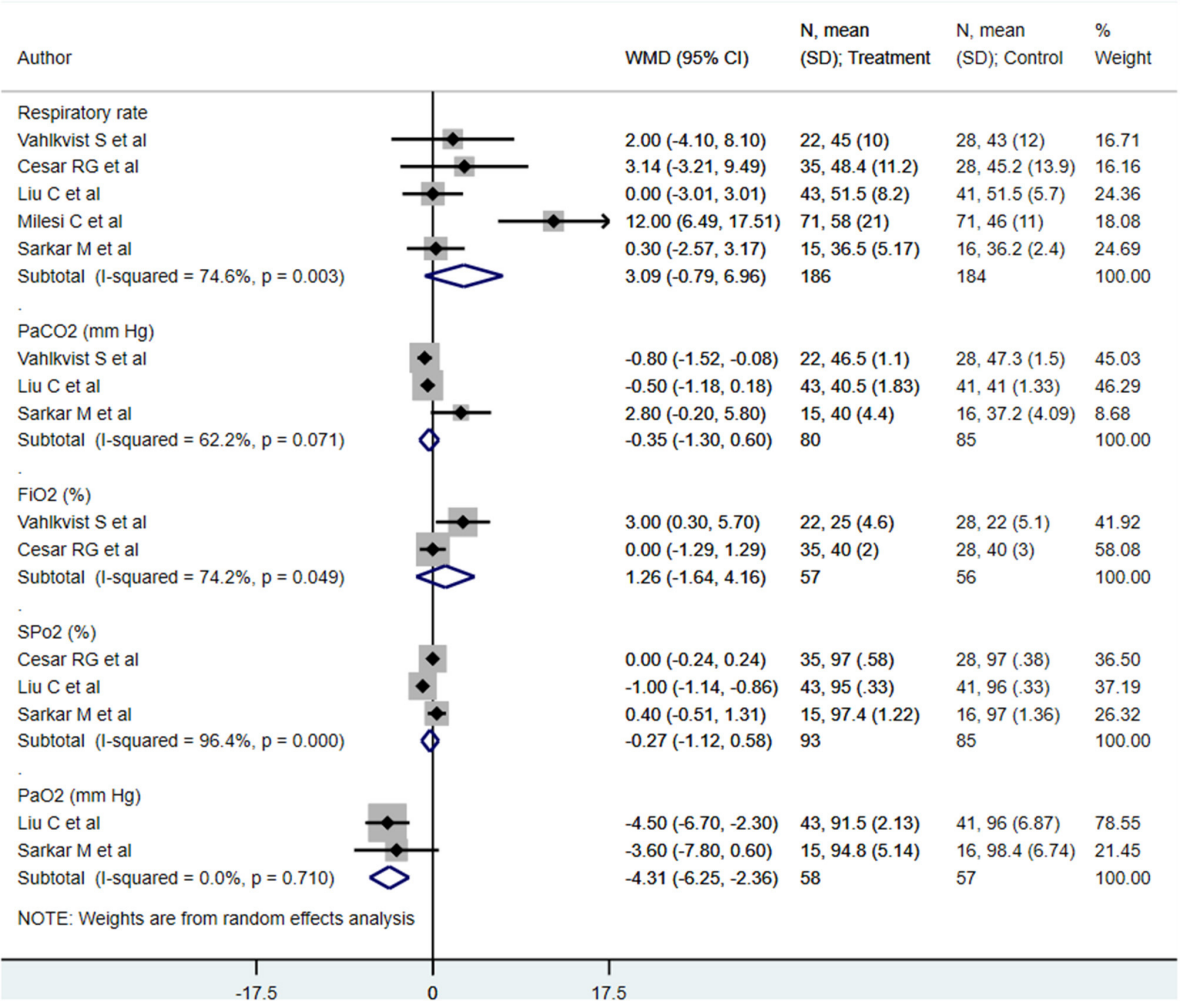
Effect of HFNC vs. CPAP on respiratory rate, PaCO_2_, FiO_2_, SpO_2_, and PaO_2_.

We found similar respiratory rates (min) [WMD, 3.09; 95% CI, −0.79 to 6.96; *I*^2^ = 74.6%, *n* = 5], PaCO_2_s (mm Hg) [WMD, −0.35; 95% CI, −1.30 to 0.60; *I*^2^ = 62.2%, *n* = 3], FiO_2_s (%) [WMD, 1.26; 95% CI, −1.64 to 4.16; *I*^2^ = 74.2%, *n* = 2], and SpO_2_s (mm Hg) [WMD, −0.27; 95% CI, −1.12 to 0.58; *I*^2^ = 96.4%, *n* = 3] at end of treatment between both treatment modalities (Figure 4). The PaO_2_ (mm Hg) at the end of treatment in patients receiving HFNC was approximately 4 mm Hg lower than that in in patients receiving CPAP [WMD, −4.31; 95% CI, −6.25 to −2.36; *I*^2^ = 0.0%, *n* = 2] (Figure 4). Egger's test results did not indicate the presence of publication bias (*P* = 0.91 for respiratory rate; *P* = 0.22 for PaCO_2_, *P* = 0.45 for FiO_2_, *P* = 0.15 for SpO_2_, and *P* = 0.39 for PaO_2_).

## Discussion

We designed this meta-analysis to compare HFNC and CPAP outcomes in pediatric patients with respiratory distress. We found that children who received HFNC treatment, compared to those that received CPAP, had significantly higher risks of treatment failure, and possibly, a higher risk for need of intubation, mortality and increased primary treatment duration. It should however be noted that there was only one study reporting on mortality and therefore, this finding should be interpreted cautiously. Patients receiving HFNC had a reduced time to treatment failure as compared to those on CPAP. This should we viewed in the context of another finding that the risk of treatment failure was also higher in those receiving HFNC. It could be possible that the higher risk of treatment failure in HFNC group necessitated early intubation, thereby, reducing the time to treatment failure. High-flow nasal cannula treatment was associated with a lower risk of adverse events than CPAP treatment. The meta-analysis results also suggested that the M-WCAS (denoting severity of respiratory distress), hospitalization lengths, respiratory rates (per min), and blood gas values [PaCO_2_ (mm Hg), FiO_2_ (%), and SpO_2_ (mm Hg)] at the end of treatment were similar between the patients receiving either treatment modality.

Acute viral bronchiolitis is a common cause of hospitalization among young children (especially in those younger than 2 years and infants) (41, 42). Continuous positive airway pressure is a common treatment in developed countries for children with respiratory distress. Continuous positive airway pressure is believed to act by decreasing the inspiratory resistance, thereby improving alveolar ventilation (25, 43). High-flow nasal cannula is an alternative technique management of respiratory distress in children, in which warm and humidified air is provided through a PEEP force (44). High-flow nasal cannula is thought to reduce the dead space in the upper airway and the airflow resistance (45). In addition, HFNC is easier to use, better tolerated by patients, and comparatively less invasive than CPAP.

Our findings are similar to those of a previous meta-analysis by Luo et al., wherein HFNC treatment was found to carry an increased risk of treatment failure and a lower risk of nasal trauma than CPAP treatment (30). Our findings are also similar to those of a meta-analysis conducted in preterm infants, which included 21 studies with approximately 3,000 preterm infants (46). That review noted that the risk of nasal trauma was lower in infants receiving HFNC than in those receiving CPAP, and that the rate of treatment failure was higher after HFNC than after CPAP. A recent systematic review by Moreel et al. looked at studies comparing HFNC with either standard oxygen therapy or CPAP among infants with bronchiolitis (47). The authors concluded that HFNC is a safe mode of respiratory support and could be considered as a rescue therapy for children who are not adequately managed on standard oxygen therapy. Only three RCTs that compared HFNC with CPAP were included in this review. Furthermore, the authors called for more RCTs comparing the efficacy of HFNC and CPAP. Similar findings were noted in another systematic review on the role of HFNC in infants with bronchiolitis (48). Thus, with the inclusion of new RCTs in our meta-analysis, the updated evidence supports the findings of the previous review on this subject. Based on findings of this meta-analysis, CPAP should remain the mainstay for providing oxygen support to children with respiratory distress from acute viral bronchiolitis and pneumonia. In situations where CPAP is not available, HFNC is a reasonable management option due to its lower adverse event rates and somewhat similar mortality when compared to those of CPAP.

An important finding of the current meta-analysis that needs further discussion is the increased risk of nasal trauma in those that received CPAP, compared to those receiving HFNC. The presentation of nasal trauma can range from erythema, crusting to scaling and excoriation of nasal mucosa (49). One of the important factors that have been implied in the increased risk of nasal trauma in CPAP is the use of nasal masks and prongs. In CPAP, there is a prerequisite that an adequate seal is maintained between the prongs and the nares and therefore, the pressure effects on nasal mucosa in unavoidable (50). On the contrary, the canula used in HFNC are shorter, narrower, tapered from base to tip and do not usually occlude more than 50% of the nares (23, 50). Consequently, the pressure effects are reduced. Further, humidification of the inspired gas in essential for preserving the integrity and intactness of the nasal mucosa. Due to improvement in the humidification systems, the humidification of the gas provided through HFNC is higher than that used in CPAP (51). This could also be one of the reasons why a lower risk of nasal trauma is noted in HFNC.

Our study has some limitations. First, the number of included studies is relatively low (*n* = 6) and provided a small sample size. More robust RCTs with adequately powered sample sizes are needed to provide conclusive evidence. Second, there was lack of homogeneity in the primary clinical diagnosis among included infants; four studies had infants with acute viral bronchiolitis and two studies included infants with pneumonia. Third, some of our secondary outcomes showed a high degree of heterogeneity among the included studies. This could be due to differences in the HFNC gas flow rates, CPAP pressures, and operational definitions for outcomes used in different studies. Finally, the included studies were conducted in varied settings and geographical areas and may have influenced the quality and intensity of care provided, the skills available for HFNC and CPAP, and the implementation of complex outcome definitions such as that of treatment failure.

## Conclusion

Nasal CPAP is associated with lower rates of treatment failure and reintubation rates but higher adverse event rates than HFNC in children with respiratory distress that required oxygen therapy. Small number of studies with limited sample size is the major limitation. Adequately powered trials are needed before issuing conclusive evidence on this issue.

## Data Availability Statement

The raw data supporting the conclusions of this article will be made available by the authors, without undue reservation.

## Author Contributions

XZhao conceived and designed the study. XZhao and QQ were involved in literature search, data collection, and wrote the paper. QQ and XZhang analyzed the data. XZhang reviewed and edited the manuscript. All authors read and approved the final manuscript.

## Conflict of Interest

The authors declare that the research was conducted in the absence of any commercial or financial relationships that could be construed as a potential conflict of interest.

## Publisher's Note

All claims expressed in this article are solely those of the authors and do not necessarily represent those of their affiliated organizations, or those of the publisher, the editors and the reviewers. Any product that may be evaluated in this article, or claim that may be made by its manufacturer, is not guaranteed or endorsed by the publisher.

## References

[1] Ghazaly MMH Abu Faddan NH Raafat DM Mohammed NA Nadel S. Acute viral bronchiolitis as a cause of pediatric acute respiratory distress syndrome. Eur J Pediatr. (2021) 180:1229–34. 10.1007/s00431-020-03852-9 33161501 PMC7648537

[2] Friedman ML Nitu ME. Acute respiratory failure in children. Pediatr Ann. (2018) 47:e268–73. 10.3928/19382359-20180625-01 30001440

[3] Meissner HC. Viral bronchiolitis in children. N Engl J Med. (2016) 374:62–72. 10.1056/NEJMra1413456 26735994

[4] Pierce HC Mansbach JM Fisher ES Macias CG Pate BM Piedra PA . Variability of intensive care management for children with bronchiolitis. Hosp Pediatr. (2015) 5:175–84. 10.1542/hpeds.2014-0125 25832972

[5] Walker CLF Rudan I Liu L Nair H Theodoratou E Bhutta ZA . Global burden of childhood pneumonia and diarrhoea. Lancet. (2013) 381:1405–16. 10.1016/S0140-6736(13)60222-6 23582727 PMC7159282

[6] World Health Organization. Pocket Book of Hospital Care for Children: Second edition. WHO Available online at: http://www.who.int/maternal_child_adolescent/documents/child_hospital_care/en/ (accessed May 19, 2021).

[7] Duke T Wandi F Jonathan M Matai S Kaupa M Saavu M . Improved oxygen systems for childhood pneumonia: a multihospital effectiveness study in Papua New Guinea. Lancet. (2008) 372:1328–33. 10.1016/S0140-6736(08)61164-2 18708248

[8] Ralston SL Lieberthal AS Meissner HC Alverson BK Baley JE Gadomski AM . Clinical practice guideline: the diagnosis, management, and prevention of bronchiolitis. Pediatrics. (2014) 134:e1474–502. 10.1542/peds.2014-2742 25349312

[9] World Health Organization. Oxygen Therapy for Children: A Manual for Health Workers. World Health Organization (2016). Available online at: https://apps.who.int/iris/handle/10665/204584 (accessed May 19, 2021).

[10] Franklin D Babl FE Schlapbach LJ Oakley E Craig S Neutze J . A randomized trial of high-flow oxygen therapy in infants with bronchiolitis. N Engl J Med. (2018) 378:1121–31. 10.1056/NEJMoa1714855 29562151

[11] Lee JH Rehder KJ Williford L Cheifetz IM Turner DA. Use of high flow nasal cannula in critically ill infants, children, and adults: a critical review of the literature. Intensive Care Med. (2013) 39:247–57. 10.1007/s00134-012-2743-5 23143331

[12] Schibler A Pham TMT Dunster KR Foster K Barlow A Gibbons K . Reduced intubation rates for infants after introduction of high-flow nasal prong oxygen delivery. Intensive Care Med. (2011) 37:847–52. 10.1007/s00134-011-2177-5 21369809

[13] Collins CL Holberton JR Barfield C Davis PG. A randomized controlled trial to compare heated humidified high-flow nasal cannulae with nasal continuous positive airway pressure postextubation in premature infants. J Pediatr. (2013) 162:949.e1–54.e1. 10.1016/j.jpeds.2012.11.016 23260098

[14] Roberts CT Owen LS Manley BJ Frøisland DH Donath SM Dalziel KM . Nasal high-flow therapy for primary respiratory support in preterm infants. N Engl J Med. (2016) 375:1142–51. 10.1056/NEJMoa1603694 27653564

[15] Essouri S Laurent M Chevret L Durand P Ecochard E Gajdos V . Improved clinical and economic outcomes in severe bronchiolitis with pre-emptive nCPAP ventilatory strategy. Intensive Care Med. (2014) 40:84–91. 10.1007/s00134-013-3129-z 24158409 PMC7095309

[16] Pinto VL Sharma S. Continuous positive airway pressure. in StatPearls. Treasure Island, FL: StatPearls Publishing. Available online at: http://www.ncbi.nlm.nih.gov/books/NBK482178/ (accessed May 19, 2021). 29489216

[17] Pedersen MB Vahlkvist S. Comparison of CPAP and HFNC in management of bronchiolitis in infants and young children. Children (Basel). (2017) 4:E28. 10.3390/children4040028 28425965 PMC5406687

[18] Yoder BA Stoddard RA Li M King J Dirnberger DR Abbasi S. Heated, humidified high-flow nasal cannula versus nasal CPAP for respiratory support in neonates. Pediatrics. (2013) 131:e1482–1490. 10.1542/peds.2012-2742 23610207

[19] Hasan RA Habib RH. Effects of flow rate and airleak at the nares and mouth opening on positive distending pressure delivery using commercially available high-flow nasal cannula systems: a lung model study. Pediatr Crit Care Med. (2011) 12:e29–33. 10.1097/PCC.0b013e3181d9076d 20228687

[20] Sivieri EM Gerdes JS Abbasi S. Effect of HFNC flow rate, cannula size, and nares diameter on generated airway pressures: an *in vitro* study. Pediatr Pulmonol. (2013) 48:506–14. 10.1002/ppul.22636 22825878

[21] Dysart K Miller TL Wolfson MR Shaffer TH. Research in high flow therapy: mechanisms of action. Respir Med. (2009) 103:1400–5. 10.1016/j.rmed.2009.04.007 19467849

[22] Hough JL Pham TMT Schibler A. Physiologic effect of high-flow nasal cannula in infants with bronchiolitis. Pediatr Crit Care Med. (2014) 15:e214–219. 10.1097/PCC.0000000000000112 24705569

[23] Mikalsen IB Davis P Øymar K. High flow nasal cannula in children: a literature review. Scand J Trauma Resusc Emerg Med. (2016) 24:93. 10.1186/s13049-016-0278-4 27405336 PMC4942966

[24] King MS Xanthopoulos MS Marcus CL. Improving positive airway pressure adherence in children. Sleep Med Clin. (2014) 9:219–34. 10.1016/j.jsmc.2014.02.003 24910579 PMC4042088

[25] Gupta S Donn SM. Continuous positive airway pressure: physiology and comparison of devices. Semin Fetal Neonatal Med. (2016) 21:204–11. 10.1016/j.siny.2016.02.009 26948884

[26] Metge P Grimaldi C Hassid S Thomachot L Loundou A Martin C . Comparison of a high-flow humidified nasal cannula to nasal continuous positive airway pressure in children with acute bronchiolitis: experience in a pediatric intensive care unit. Eur J Pediatr. (2014) 173:953–8. 10.1007/s00431-014-2275-9 24525672

[27] ten Brink F Duke T Evans J. High-flow nasal prong oxygen therapy or nasopharyngeal continuous positive airway pressure for children with moderate-to-severe respiratory distress?^*^. Pediatr Crit Care Med. (2013) 14:e326–31. 10.1097/PCC.0b013e31828a894d 23842586

[28] Manley BJ Owen LS Doyle LW Andersen CC Cartwright DW Pritchard MA . High-flow nasal cannulae in very preterm infants after extubation. N Engl J Med. (2013) 369:1425–33. 10.1056/NEJMoa1300071 24106935

[29] Murki S Singh J Khant C Kumar Dash S Oleti TP Joy P . High-flow nasal cannula versus nasal continuous positive airway pressure for primary respiratory support in preterm infants with respiratory distress: a randomized controlled trial. Neonatology. (2018) 113:235–41. 10.1159/000484400 29393237

[30] Luo J Duke T Chisti MJ Kepreotes E Kalinowski V Li J. Efficacy of high-flow nasal cannula vs standard oxygen therapy or nasal continuous positive airway pressure in children with respiratory distress: a meta-analysis. J Pediatr. (2019) 215:199–208.e8. 10.1016/j.jpeds.2019.07.059 31570155

[31] Lin J Zhang Y Xiong L Liu S Gong C Dai J. High-flow nasal cannula therapy for children with bronchiolitis: a systematic review and meta-analysis. Arch Dis Child. (2019) 104:564–76. 10.1136/archdischild-2018-315846 30655267

[32] Page MJ McKenzie JE Bossuyt PM Boutron I Hoffmann TC Mulrow CD . The PRISMA 2020 statement: an updated guideline for reporting systematic reviews. BMJ. (2021) 372:n71. 10.1136/bmj.n71 33782057 PMC8005924

[33] Higgins JPT Altman DG Gøtzsche PC Jüni P Moher D Oxman AD . The Cochrane Collaboration's tool for assessing risk of bias in randomised trials. BMJ. (2011) 343:d5928. 10.1136/bmj.d5928 22008217 PMC3196245

[34] Higgins J Green S. Cochrane Handbook for Systematic Reviews of Interventions. Cochrane Collaboration (2009). 10.1002/9780470712184

[35] Vahlkvist S Jürgensen L. la Cour A, Markoew S, Petersen TH, Kofoed P-E. High flow nasal cannula and continuous positive airway pressure therapy in treatment of viral bronchiolitis: a randomized clinical trial. Eur J Pediatr. (2020) 179:513–8. 10.1007/s00431-019-03533-2 31828528

[36] Cesar RG Bispo BRP Felix PHCA Modolo MCC Souza AAF Horigoshi NK . High-flow nasal cannula versus continuous positive airway pressure in critical bronchiolitis: a randomized controlled pilot. J Pediatr Intensive Care. (2020) 9:248–55. 10.1055/s-0040-1709656 33133739 PMC7588294

[37] Liu C Cheng WY Li JS Tang T Tan PL Yang L. High-flow nasal cannula vs. continuous positive airway pressure therapy for the treatment of children <2 years with mild to moderate respiratory failure due to pneumonia. Front Pediatr. (2020) 8:590906. 10.3389/fped.2020.590906 33304868 PMC7693448

[38] Chisti MJ Salam MA Smith JH Ahmed T Pietroni MAC Shahunja KM . Bubble continuous positive airway pressure for children with severe pneumonia and hypoxaemia in Bangladesh: an open, randomised controlled trial. Lancet. (2015) 386:1057–65. 10.1016/S0140-6736(15)60249-5 26296950

[39] Milési C Essouri S Pouyau R Liet J-M Afanetti M Portefaix A . High flow nasal cannula (HFNC) versus nasal continuous positive airway pressure (nCPAP) for the initial respiratory management of acute viral bronchiolitis in young infants: a multicenter randomized controlled trial (TRAMONTANE study). Intensive Care Med. (2017) 43:209–16. 10.1007/s00134-016-4617-8 28124736

[40] Sarkar M Sinha R Roychowdhoury S Mukhopadhyay S Ghosh P Dutta K . Comparative study between noninvasive continuous positive airway pressure and hot humidified high-flow nasal cannulae as a mode of respiratory support in infants with acute bronchiolitis in pediatric intensive care unit of a tertiary care hospital. Indian J Crit Care Med. (2018) 22:85–90. 10.4103/ijccm.IJCCM_274_17 29531447 PMC5842462

[41] Piedimonte G Perez MK. Respiratory syncytial virus infection and bronchiolitis. Pediatr Rev. (2014) 35:519–30. 10.1542/pir.35-12-519 25452661 PMC5029757

[42] Nair H Nokes DJ Gessner BD Dherani M Madhi SA Singleton RJ . Global burden of acute lower respiratory infections due to respiratory syncytial virus in young children: a systematic review and meta-analysis. Lancet. (2010) 375:1545–55. 10.1016/S0140-6736(10)60206-1 20399493 PMC2864404

[43] Essouri S Durand P Chevret L Balu L Devictor D Fauroux B . Optimal level of nasal continuous positive airway pressure in severe viral bronchiolitis. Intensive Care Med. (2011) 37:2002–7. 10.1007/s00134-011-2372-4 21993811

[44] Pham TMT O'Malley L Mayfield S Martin S Schibler A. The effect of high flow nasal cannula therapy on the work of breathing in infants with bronchiolitis. Pediatr Pulmonol. (2015) 50:713–20. 10.1002/ppul.23060 24846750

[45] Milési C Baleine J Matecki S Durand S Combes C Novais ARB . Is treatment with a high flow nasal cannula effective in acute viral bronchiolitis? A physiologic study. Intensive Care Med. (2013) 39:1088–94. 10.1007/s00134-013-2879-y 23494016

[46] Hong H Li X-X Li J Zhang Z-Q. High-flow nasal cannula versus nasal continuous positive airway pressure for respiratory support in preterm infants: a meta-analysis of randomized controlled trials. J Matern Fetal Neonatal Med. (2021) 34:259–66. 10.1080/14767058.2019.1606193 30966839

[47] Moreel L Proesmans M. High flow nasal cannula as respiratory support in treating infant bronchiolitis: a systematic review. Eur J Pediatr. (2020) 179:711–8. 10.1007/s00431-020-03637-0 32232547

[48] Fainardi V Abelli L Muscarà M Pisi G Principi N Esposito S. Update on the role of high-flow nasal cannula in infants with bronchiolitis. Children (Basel). (2021) 8:66. 10.3390/children8020066 33498527 PMC7909574

[49] Shanmugananda K Rawal J. Nasal trauma due to nasal continuous positive airway pressure in newborns. Arch Dis Child Fetal Neonatal Ed. (2007) 92:F18. 10.1136/adc.2006.095034 17185425 PMC2675289

[50] Collins CL Barfield C Horne RSC Davis PG. A comparison of nasal trauma in preterm infants extubated to either heated humidified high-flow nasal cannulae or nasal continuous positive airway pressure. Eur J Pediatr. (2014) 173:181–6. 10.1007/s00431-013-2139-8 23955516

[51] Chang GY Cox CA Shaffer TH. Nasal cannula, CPAP, and high-flow nasal cannula: effect of flow on temperature, humidity, pressure, and resistance. Biomed Instrum Technol. (2011) 45:69–74. 10.2345/0899-8205-45.1.69 21322815

