# Supplementary material for: Outcomes of High-Flow Nasal Cannula Vs. Nasal Continuous Positive Airway Pressure in Young Children With Respiratory Distress: A Systematic Review and Meta-Analysis
Source: Front Pediatr. 2021 Nov 5;9:759297. doi: 10.3389/fped.2021.759297 (PMC8602879; doi:10.3389/fped.2021.759297)
Supplement: Supplementary file 1 [file Data_Sheet_1.doc]

**Supplemental table 1. Search strategy for identification of studies to be included in the review**

| **Search strategy**  #1 (High-Flow Nasal Cannula OR Nasal Continuous Positive Airway Pressure)  #2 (Pediatrics OR children OR young infants)  #3 (Respiratory distress OR acute bronchiolitis OR pulmonary distress OR lung disease)  #4 (#1 AND #2 AND #3)  #5 (Addresses[ptyp] OR Autobiography[ptyp] OR Bibliography[ptyp] OR Biography[ptyp] OR pubmed books[filter] OR Case Reports[ptyp] OR Congresses[ptyp] OR Consensus Development Conference[ptyp] OR Directory[ptyp] OR Duplicate Publication[ptyp] OR Editorial[ptyp] OR Systematic reviews OR Meta analysis OR Festschrift[ptyp] OR Guideline[ptyp] OR In Vitro[ptyp] OR Interview[ptyp] OR Lectures [ptyp] OR Legal Cases[ptyp] OR News[ptyp] OR Newspaper Article[ptyp] OR Personal Narratives [ptyp] OR Portraits[ptyp] OR Retracted Publication[ ptyp] OR Twin Study[ptyp] OR Video-Audio Media[ptyp])  #6 (#4 NOT #5) |
| --- |

**Supplementary table 2. Author’s judgements about each risk of bias for each included study based on Cochrane risk of bias assessment items**

| **Included studies** | **Random sequence generation** | **Allocation concealment** | **Blinding of participants*** | **Blinding of personnel*** | **Blinding of outcome assessment team** | **Attrition bias** | **Other bias** |
| --- | --- | --- | --- | --- | --- | --- | --- |
| Vahlkvist S et al (2019) | + | + | NA | NA | NA | - | None identified |
| Cesar RG et al (2020) | + | + | NA | NA | NA | - | None identified |
| Liu C et al (2020) | + | + | NA | NA | NA | ? | None identified |
| Chisti MJ et al (2015) | + | + | NA | NA | NA | - | None identified |
| Milesi C et al (2017) | + | + | NA | NA | NA | - | None identified |
| Sarkar M et al (2018) | + | + | NA | NA | NA | ? | None identified |

*NA as the trials were open label and therefore blinding could not be ensured; + denotes presence; ? denotes unclear; - denotes absence
